# Life and the Municipality

**Published:** 1896-07-18

**Authors:** 


### The Hospital

An Institutional Journal of

The Medical Sciences and Hospital Administration, with

AN EXTRA NURSING SUPPLEMENT.

Vol. XX.—No. 512] [July 18, 1896.

### Life and the Municipality.

Martial wrote with force and truth that life is not to live but to be well. During the week which has just passed the principles contained in Martial's epigram have been forcibly driven home by various speakers, and especially by Mr. Chamberlain, M.P , at the banquets which have been held in honour of the municipal ideal. There can be no doubt that life in the highest and best sense of that term has been much promoted by the great municipal movement which has taken place in this country during the last forty years. As Mr. Chamberlain well said, "If among the many cities of the United Kingdom one only would have been patriotic enough to remain perpetually in its pristine state of dirt, discomfort, and disease, what a fine moral, social, and political lesson a pilgrimage to that town would have been." Fifty years ago municipal activities were undeveloped. At that time residence in a large town in this country was attended with dangers to health and even to life, owing to bad government and worse sanitation, which the present generation cannot even conceive, much less realise. To live in a town half a century ago was certainly not to be well, and as surely may it be said that living in those days was not to live but merely to exist. It is well occasionally to recall the past, because nowadays most people live at such a pace that they pay little regard to its teachings and give no time for reflection, which must otherwise enable them to cultivate a feeling of thankfulness for the present.

The greatest good of the greatest number should ever be the aim of all politicians, both local and imperial. Parliament has continuously conferred additional powers upon municipalities in this country until local self-government has become to-day so important a feature of our national life that it affords honour to and an outlet for the best energies of all classes of citizens from the highest to the humblest. Municipalities now own the gas works, the water supply, and a large and increasing number of the houses occupied by the artisan class. They expend money in the provision of baths and washhouses, the opening and maintenance of public parks and playing fields for the people, and the provision of bands and other things which largely contribute to the enjoyment and light-heartedness, and consequently to the health and well-being, of the citizens. Further than this, they encourage intellectual development and manual skill by the maintenance of public libraries and art galleries, and by the support of technical and secondary schools, which afford the fullest opportunities for the acquirement of knowledge and skill, the possession of which must contribute largely to the earning power and intelligence of those who avail themselves of such aids to self-education and advancement.

If the wave of municipal movement which began to manifest itself in this country some thirty years ago had accomplished no more than the results here briefly referred to, it must have established its claim to be regarded as one of the most noticeable and influential factors in the development of the well-being of the people during the present century. The rise of the municipality has, however, had even a deeper and a higher meaning by ensuring that those who occupy the lowest grade in the social scale of town life shall be given an opportunity and a hope. The unskilled workman and the lower and poorest classes of town residents, which represent about one-sixteenth of the total population, were sadly neglected in every respect fifty years ago. Nowadays their utility and value to the well-to-do, and indeed to all classes of citizens in towns, is fully recognised. Formerly it was impossible for the unskilled workman and the poorer classes in towns to obtain decent house accommodation for a rental within the means of their scanty earnings. The municipality in many of our large towns has altered all this by teaching the people that every citizen has a right to take part in municipal life, and that the needs and interests of the humblest classes must be met by the municipality as the authority which represents the people. Hence free education has now been placed within the reach of every ratepayer who requires it, and the municipalities and county councils have systematically worked with the object of securing the destruction and extinction of the slums, and the substitution for them of decent houses for the occupation of the poorest classes of the inhabitants who have been largely exempted from rates, and, where necessary, pro vided with free water and free gas. We are distinctly of opinion that the municipal idea will not have attained perfection until we have secured that the municipality shall own a sufficient number of houses, exempted from rates and provided with free water and light, which can be let on a business basis to the unskilled working man and the lower and poorest classes of towns at a rental which shall not exceed one-eighth of the weekly earnings of the occupiers of this class of property. The municipalities can borrow money cheaper than anyone else, say at $2\frac{1}{2}$ per cent., and it has been shown that the class of house property we refer to, when owned by the local authority, can be made to return at the rentals just given a net profit of about 4 per cent. It follows that every municipality which properly acts up to its responsibilities in this matter will be able to so adjust the financial part of this scheme that they can repay the principal sum in a term of years by means of a sinking fund, and adjust the prices of sites in addition out of the profit of $1\frac{1}{2}$ per cent. per annum, which the proposals here referred to would yield.

It is right and proper, therefore, that Mr. Chamberlain, Lord Rosebery, and others should toast the municipal idea and advocate its extension and development throughout the country. We owe the diminution in the death rate, the improvement in the general health and physique of the people, their greater happiness, increased intelligence, and growing power of work and activity, largely to the municipal movement. In the corporations and county councils of this country the young men of all classes acquire a sound knowledge of business of all kinds, and especially of public business, and the absence of this training or its possession makes all the difference in the position which a man is able to take in the House of Commons should he find a constituency willing to return him to take part in the councils of the nation.

All honour, then, to those responsible for municipal administration and the management of the local government of our large towns. The intelligence they have displayed, and their readiness in responding to the opportunities afforded them by Parliament to improve the condition of the people everywhere, has enabled hundreds and thousands of the population to live who formerly found it hard to exist. The medical profession has welcomed this awakening, and has not failed to aid the movement by educating the people in the principles of public health and the teachings of science, so that the outlook for residents in towns in this country to-day is bright with hope and full of promise for the future.

